# Is gynaecological surgical training a cause for concern? A questionnaire survey of trainees and trainers

**DOI:** 10.1186/1472-6920-11-32

**Published:** 2011-06-13

**Authors:** Esther L Moss, Foteini E Bredaki, Peter W Jones, James Hollingworth, David M Luesley, Kiong K Chan

**Affiliations:** 1 Pan Birmingham Gynaecological Cancer Centre, West Midlands, UK; 2 Instutute of Science and Technology in Medicine, Keele University, Staffordshire, UK; 3 Department of Obstetrics and Gynaecology, Queens Hospital, Burton on Trent, UK

## Abstract

**Astract:**

**Background**

Concerns have been raised as to whether the current postgraduate training programme for gynaecological surgery is being detrimentally affected by changes in working practices, in particular the European Working Time Directive (EWTD). The purpose of this study was to investigate the surgical activity of obstetrics and gynaecology trainees and to explore trainees' and trainers' opinions on the current barriers and potential solutions to surgical training.

**Methods**

Two questionnaire surveys were conducted, one to obstetrics and gynaecology trainees working within the West Midlands Deanery and a second to consultant gynaecologists in the West Midlands region.

**Results**

One hundred and four trainees (64.3%) and 66 consultant gynaecologists (55.0%) responded. Sixty-six trainees (66.7%) reported attending up to one operating list per week. However, 28.1% reported attending up to one list every two weeks or less and 5 trainees stated that they had not attended a list at all over the preceding 8 weeks. Trainees working in a unit with less than 3999 deliveries attended significantly more theatre sessions compared to trainees in units with over 4000 deliveries (p = 0.007), as did senior trainees (p = 0.032) and trainees attached to consultants performing major gynaecological surgery (p = 0.022). In the previous 8 weeks, only 6 trainees reported performing a total abdominal hysterectomy independently, all were senior trainees (ST6 and above). In the trainers' survey, only two respondents (3.0%) agreed that the current program produces doctors competent in general gynaecological surgery by the end of training, compared to 48 (73.8%) respondents who disagreed.

**Conclusions**

Trainees' concerns over a lack of surgical training appear to be justified. The main barriers to training are perceived to be a lack of team structure and a lack of theatre time.

## Background

The challenge of acquiring sufficient surgical skills in a reduced training time in order to function safely at consultant level is not a problem confined to gynaecology and applies to all specialties where trainees need to acquire practical skills[1-4]. The traditional view of obstetrics and gynaecology as one specialty appears increasingly problematic due to subspecialisation, along with a shortened training program and the implementation of the European Working Time Directive (EWTD). A need to provide general exposure to the specialty has raised concerns that gynaecological surgical training in particular is being detrimentally affected. The specific causes of reduced training opportunities and their potential solutions are the source of much dissatisfaction amongst both trainees and trainers[5], exacerbated by the inevitable uncertainties surrounding workforce planning.

The aim of this study was to determine the current viewpoint of gynaecological surgical training in the West Midlands (WM) from the perspective of both trainees and trainers (consultants).

## Methods

An internet-based questionnaire using the survey tool 'Surveymonkey' was designed to investigate the surgical activity and self reported confidence of obstetrics and gynaecology trainees. The UK postgraduate specialty training (ST) programme is divided into basic training (ST1 and ST2), intermediate training (ST3, ST4 and ST5) and advanced training (ST6 and ST7), with the ST number usually reflecting the number of years a trainee has spent in the specialty. The survey contained questions exploring trainee's opinions on current barriers and potential solutions to surgical training (Additional file 1). The questionnaires had not been previously validated but were piloted by the WM Trainees' Committee and five consultant gynaecologists. The questionnaire to the trainees was distributed to all the trainees on the WM Deanery's email list, three reminder emails were sent and the survey link was kept open for 4 weeks. At the time of the survey there were 183 trainees in the WM region, of whom 16 were on maternity leave and 12 on out of programme training (OOPT). Trainees on maternity leave or on OOPT were excluded from the study, except for the trainees in subspecialty training posts. This gave a potential survey population of 157.

A second questionnaire investigating the views of consultant gynaecologists on current surgical training was designed, piloted and distributed in a similar manner (Additional file 2). A list of consultants working as gynaecologists in the WM was obtained by contacting the individual hospitals directly and an email containing a link to the survey sent to the consultants' hospital email address. One reminder email was sent after 3 weeks and the link kept open for 6 weeks.

Trainees were asked how many days leave (annual/study/sick) they had taken over the past 8 weeks. Assuming a 5 day week, the number of days the trainee had worked over the previous 8 weeks was totaled and the number of theatre sessions attended per week calculated. Ordered logistic regression models were fitted using the Intercooled Stata 9.2 (Stata Corp LP, College Station TX, 2007) program in order to determine an association between the number of operating lists attended and the predictors: the size of the unit (<3999, 4000-5999, >6000 deliveries), seniority (ST1-2, ST3-5, ST6^+^) and the specialty of the trainees' supervising consultant (obstetrics only/minor gynaecological surgery, major gynaecological surgery). A stepdown procedure was carried out to find the best subset of predictors. For the purposes of analysis and comparison with previous work, the operation of total abdominal hysterectomy with or without ovarian conservation (TAH) was used as an example of a general operation in gynaecology because this requires a range of surgical skills, is undertaken by all units and is an essential requirement of the core log-book.

## Results

One hundred and four trainees responded to the questionnaire, three were excluded due to maternity leave or currently being in a research post, giving a response rate of 64.3% (Table 1). Sixty-six out of the 120 consultants who were successfully emailed responded to the questionnaire, giving a response rate of 55.0% (Table 2).

**Table 1:** Characteristics of the trainees responding to the questionnaire, n = 101

	Number of respondents (%)
Seniority	
ST 1-2	31 (30.7)
ST 3-5	48 (47.5)
ST 6^+ ^(including SST/post CCT)	22 (21.8)
Membership	
MRCOG part 1	36 (35.6)
MRCOG part 2	49 (48.5)
Career intentions	
Obstetrics only	10 (9.9)
Gynaecology only	14 (13.9)
Both - minor gynaecological surgery	15 (14.9)
Both - major gynaecological surgery	39 (38.6)
Undecided/not stated	23 (22.8)
Number of deliveries per annum	
<3999	42 (41.6)
4000-5999	35 (34.7)
>6000	24 (23.8)
Current consultant attachment	
Obstetrics only/Minor gynaecological surgery	15 (14.9)
Major gynaecological surgery	86 (85.1)

**Table 2:** Characteristics of the consultant gynaecologists responding to the questionnaire, n = 66

	Number of respondents (%)
Specialty	
Obstetrics with minor gynaecological surgery	3 (4.5)
Obstetrics with major gynaecological surgery	46 (69.7)
Gynaecology only	17 (25.8)
Expertise	
Urogynaecology	9 (13.6)
Infertility	4 (6.1)
Benign gynaecology	32 (48.5)
Oncology - unit	12 (18.2)
Oncology - centre	9 (13.6)
Number of years at consultant level	
<5 years	16 (24.2)
5-14 years	33 (50.0)
>15 years	17 (25.8)
Number of deliveries per annum	
<3999	28 (45.2)
4000-5999	20 (32.2)
>6000	14 (22.6)

Concern was expressed by both the trainees and trainers over the current gynaecological surgical training. Only a minority of trainees reported that they thought they were getting enough surgical experience (18.8%) or teaching (20.8%). In the consultants' survey, only two respondents (3.0%) agreed that the current program produces doctors competent in general gynaecological surgery by the end of training, compared to 48 (73.8%) respondents who disagreed. There was also a lack of confidence in the methods used for assessment with only 15.1% of consultants agreeing with the statement 'OSATS (objective structured assessment of technical skill) are a genuine measure of competence'.

**Figure 1:**
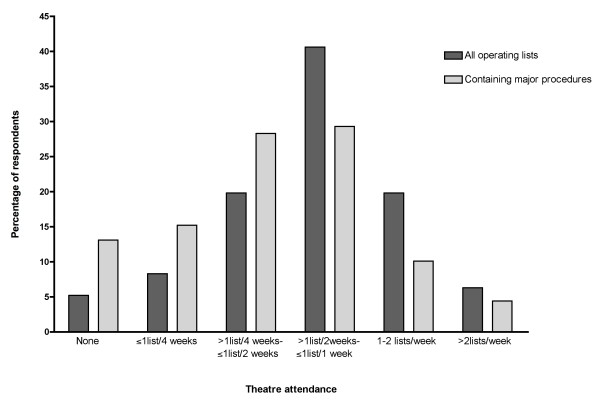
**Reported theatre attendance by trainees over the previous eight weeks, n = 101**.

Sixty-seven percent of trainees reported attending up to one or more operating list per week, however, 28.1% reported attending up to one list every two weeks or less and five stated that they had not attended a list at all over the preceding 8 weeks (Figure 1). Multivariate analysis of the number of sessions attended revealed that trainees working in a unit with less than 3999 deliveries attended significantly more theatre sessions compared to trainees in units with 4000-5999 and over 6000 deliveries, odds ratio (OR) 2.99 p = 0.007 (95% CI 1.34-6.67). Senior trainees (ST6^+^) attended more sessions OR 2.84 p = 0.032 (95% CI 1.09-7.38), as did trainees who were attached to consultants who performed major gynaecological surgery, OR 3.61 p = 0.022 (95% CI 1.21-10.79). When the number of operating lists including major procedures was examined, trainees attached to a consultant performing major surgery attended significantly more lists compared to trainees attached to obstetricians or those only performing minor procedures, OR 3.92 p = 0.017 (95% CI 1.28-12.06). Trainees based in a hospital delivering 4000-5999 per year attended significantly less major lists compared to the size other units, OR 0.33 p = 0.006 (95% CI 0.15-0.72). Whether the trainee had obtained the MRCOG has no significant effect for either dependent variable.

In the previous 8 weeks, only 6 trainees reported performing a TAH independently, all were ST6 and above. Twenty-one (43.8%) ST3-5s and 14 (63.6%) ST6^+^s reported being the primary surgeon for at least one TAH during that time period. When the trainees were asked which procedures they felt confident to perform independently only 12.1% of ST3-5s and 84.2% of ST6^+^s reported being confident to perform a TAH (Table 3).

**Table 3:** Trainees' surgical confidence

	ST1-2	ST3-5	**ST6**^**+**^
Total abdominal hysterectomy	0/18 (0)	4/33 (12.1)	16/19 (84.2)
Vaginal hysterectomy	0/18 (0)	2/33 (6.1)	12/17 (70.6)
Laparoscopic hysterectomy	0/18 (0)	0/32 (0)	2/17 (11.8)
Diagnostic laparoscopy	6/20 (30.0)	41/45 (91.1)	20/20 (100)
Laparoscopic ectopic	1/19 (5.3)	13/38 (34.2)	17/19 (89.5)
Vaginal repair	2/19 (10.5)	11/36 (30.6)	19/20 (95.0)
Diagnostic hysteroscopy	16/21 (76.2)	47/47 (100)	20/20 (100)
Myomectomy	1/18 (5.6)	2/32 (6.3)	13/18 (72.2)

The procedures trainees reported to be happy to perform independently. Figure in brackets is the percentage. N = 101, not all trainees responded to every question.

Consultants reported a median of two different trainees attending their major lists over the preceding 4 weeks, with a range from no trainees (five responses) up to 8 different trainees. Six consultants (9.1%) reported always having more than one trainee in theatre at a time, 17 (25.8%) answered regularly and 35 (53.0%) rarely. Having more than one trainee in theatre at a time was not associated with the number of deliveries at the unit (p = 0.86).

The views of the trainees and trainers on the potential barriers to surgical training were similar, with both groups identifying a lack of continuity between consultant/trainee and a lack of theatre time as being major factors. Trainees also indicated that staff shortages preventing them from attending theatre were also a major problem (Table 4 and 5). Of the free comments entered by the consultants 48.6% indicated that EWTD/48 hour working week was a particular barrier to training. This reason was also mentioned by trainees but their responses also highlighted rota inequalities and the prioritisation of obstetric cover over gynaecological surgery as significant problems.

**Table 4:** Trainees' opinions of potential barriers to gynaecological surgical training

	Yes/definitely Yes	Unsure	No/Definitely not
Unable to attend theatre due to staff shortages	79 (83.2)	3 (3.2)	13 (13.7)
Unable to attend theatre due to time off following on calls	74 (78.7)	9 (9.6)	11 (11.7)
Lack of theatre lists to attend	39 (44.3)	21 (23.9)	28 (31.8)
Lack of theatre time to allow training	74 (80.4)	9 (9.8)	9 (9.8)
Consultant not surgically confident to teach	31 (34.4)	13 (14.4)	46 (51.1)
Consultant not inclined to teach	45 (48.9)	10 (10.9)	37 (40.2)
Lack of continuity with the same consultant	67 (72.8)	10 (10.9)	15 (16.3)

Figure in brackets is percentage. N = 101, not all trainees responded to every question.

**Table 5:** Consultant gynaecologists' opinions on potential barriers to surgical training

	Yes definitely/Yes	Unsure	No/Definitely not
Lack of continuity with same trainee	60 (90.0)	1 (1.5)	5 (7.5)
Lack of theatre time to allow training	55 (83.3)	2 (3.0)	9 (13.6)
Cases too complex to allow training	38 (57.6)	4 (6.1)	24 (36.3)
Operating with a consultant colleague	1 (11.0)	7 (10.9)	50 (78.2)
Lack of enthusiasm/inclination by trainee to learn	10 (15.4)	16 (24.6)	39 (60.0)
Trainees lacking basic surgical skills	30 (45.5)	14 (21.2)	22 (33.3)
Lack of interest by consultants to teach	9 (14.3)	8 (12.7)	46 (73.0)
Lack of confidence by consultants to teach	12 (18.5)	12 (18.5)	41 (63.1)

Figure in brackets is percentage. N = 66, not all trainers responded to every question.

When eliciting views on the potential solutions to the current training problems the trainees and consultants again had similar views, with the importance of attachment to a surgical trainer and the introduction of designated training lists both gaining high scores (Table 6). Both groups also understood the need for gaining skills outside the operating theatre agreeing that there should be increased use of models and laparoscopic trainers. Over half of trainees, 53.2%, reported that they were prepared to attend lists in their own time as a way of gaining more training. Finally, consultants were asked whether they thought that surgical aptitude testing should be introduced to select trainees for surgical training and the majority agreed (60.6%) with only 13.6% disagreeing.

**Table 6:** Trainees' and consultant gynaecologists' (trainers) opinions on potential solutions to the problems associated with gynaecological surgical training

	Trainees			Trainers		
	Yes	Unsure	No	Yes	Unsure	No
Attachment to a surgical trainer	91 (93.8)	3 (3.1)	3 (3.1)	58 (89.2)	7 (10.8)	0
Attachment to a gynaecological oncologist	56 (57.1)	32 (32.7)	10 (10.2)	17 (42.2)	24 (37.5)	13 (20.3)
Designated training lists	94 (96.9)	2 (2.1)	1 (1.0)	52 (80.0)	10 (15.4)	3 (4.6)
Increased use of models/laparoscopic trainers	79 (80.6)	15 (15.3)	4 (4.1)	47 (72.3)	10 (15.4)	8 (12.3)

Figure in brackets is percentage. Not all trainees or trainers responded to all the questions.

## Discussion

The aim of this study was to obtain a balanced view of current gynaecological surgical training in the West Midlands and the response rate for a questionnaire-based survey is good. There is always the possibility of responder bias in studies of this type, however, responses were received from two thirds of trainees and over a half of consultants and therefore the results can be taken as the views of the majority.

The study attempted to objectively measure trainees' attendance in theatre and the number of procedures being performed. The results showed that although 66.7% of trainees were attending up to one operating list per week, when attendance at lists containing only minor procedures was excluded this percentage fell to 43.8%. Of particular concern was the fact that 28.3% of trainees reported attending a list with a major procedure once a month or less. Attendance in gynaecological theatre is a core component of clinical training, however unlike obstetric skills, training opportunities are predominantly limited to daytime hours. The introduction of the EWTD has changed trainees' working patterns resulting in a substantial reduction in daytime working and consequentially a reduction in theatre attendance. Staff shortages can compound this reduction with trainees being removed from theatre to cover essential services, in particular the labour ward, as was highlighted in the trainees' responses. This reduction in training opportunities has been shown across many specialties[6-8] and appears to be particularly apparent in specialties with high levels of service commitment[9]. While the reduction in trainee attendance in theatre can be explained by the current working environment this does not help in finding a solution and expecting trainees to learn surgical skills with such erratic and infrequent attendance may be unrealistic.

The number of operating sessions that the trainees reported attending was significantly associated with the size of the unit, with trainees based in hospitals with less than 3999 deliveries per year able to attend theatre more regularly. Possible reasons for this may again be due to staffing issues, which may be more pronounced in larger units where greater junior cover for service is required. Also interesting to note is that trainees who are attached to consultants who did not perform major gynaecological procedures were significantly less likely to attend theatre or lists containing major cases. A consultant body with diverse expertise can allow trainees to gain specialist knowledge and training but it may not be optimal for core training. The attachment to a surgical trainer may be a potential solution for training and was popular with both trainees and trainers. Previous reports[9] have suggested that not all consultants should be obliged to teach, instead selecting for aptitude and skills, since as highlighted in this study not all consultants are confident or inclined to teach. It is also recognised that there should be support for those who do undertake such a role[9] since it is accepted that training requires time and trainees will be slower than consultants at performing procedures[10] and this is difficult to reconcile with the current pressures on waiting times and theatre utilisation. The introduction of training lists has previously been proposed as a solution and this measure gained overwhelming support by all the respondents, however, the practicalities of arranging such lists, selecting suitable patients and determining who is going to bear the financial cost is something that may prove impossible to resolve[10].

The study showed that when trainees did attend theatre the number of major procedures performed was low and very few trainees have opportunities to operate independently. This can have an impact on the transition from junior to consultant and strengthens the argument for the development of the junior consultant grade or post-CCT fellowship posts[11]. A lack of surgical confidence was highlighted in our results with only 86.4% of ST6^+ ^trainees happy to perform a TAH independently as compared to in a study in 2002[12] where the level was 99% in a similar senior population. The percentage fall may appear to be small but the difference is significant, Yates corrected Chi squared p = 0.008. When we consider that the trainees have less than 2 years to completing their training it does raise concerns as to whether they will be able to improve their skills in the remaining training time in order to function safely at consultant level. Another marker of surgical confidence was that not all senior trainees were confident to performed a laparoscopic salpingectomy for an ectopic pregnancy independently despite the RCOG guidelines stating that the laparoscopic approach is the preferred surgical route[13]. Also of concern is the low level of surgical confidence reported for the vaginal hysterectomy. Although not performed as frequently as the TAH, specific skills are required for vaginal surgery and in acknowledgement of this the RCOG has developed an advanced training skills module (ATSM) focused on benign vaginal surgery. It is difficult to determine whether the reasons for the low level of confidence in performing a vaginal hysterectomy are the same as for the TAH, however, it is probable that reduced exposure to the procedure due to consultant subspecialisation is a factor. A low level of surgical confidence is likely to be carried through to consultant level and may explain the result in Table 4 where a third of trainees reported that they felt that consultants were not surgically confident to teach, therefore perpetuating the cycle of poor training.

Reasons for not operating independently will be multi-factorial and include clinical governance issues and not being confident in or familiar with the trainees' surgical skills. Another major reason may be the dramatic decrease in the volume of routine gynaecological surgery over the past few decades, due to the introduction of less morbid alternatives, resulting in many of the patients who do require surgery having complex pathology which may not be suitable for a trainee to perform independently. Such cases may still provide many learning opportunities even if the trainee is not the primary surgeon.

Both trainees and trainers recognised the importance of gaining experience outside the operating theatre by improving technical skills using laparoscopic trainers and models. Much work has been invested into devising programmes, including postgraduate degrees, utilising such equipment[14] and the results have shown that the skills acquired through simulation training are transferable to the operating theatre[15,16]. Structured training for specific procedures using simulation equipment does shorten the learning curve for laparoscopic procedures on real patients[17,18], however, the availability of virtual reality training equipment will be limited in many units and therefore it is difficult to know how a teaching programme with simulation as its foundation will be possible for the majority of trainees in regions such as the West Midlands.

Concern was raised by trainers of the ability of the OSATS to function as an assessment tool in the everyday clinical practice as they do in examination circumstances[19]. The pressure on time in everyday clinical practice may affect the ability of trainers to thoroughly assess and feedback on a trainee's performance therefore reducing the OSAT to a paper exercise. This view by trainers may be a reflection of unhappiness with a lack of continuity in training and may support the view that a more comprehensive training/assessment programme is needed through attachment to a surgical trainer[20] or a well organised, well supervised, supportive educational environment[21].

With increased subspecialisation not all consultants perform major surgery, therefore when considering workforce planning not all juniors will require training in major surgery. Selection of trainees may involve an element of self-selection, as shown in our study with a quarter of trainees indicating that they intended their future consultant post to contain only obstetrics or minor gynaecological surgery. This study also showed support for the objective selection of surgical candidates with aptitude testing, although this may be already in place for senior trainees with competitive selection for surgical ATSMs.

## Conclusions

Trainees' concerns over a lack of surgical training appear to be justified with the majority of trainees reporting low levels of surgical activity. The main barriers to training are perceived to be a lack of team structure and a lack of theatre time. Although many of the problems can be traced back to the changes in working practice associated with the introduction of the EWTD some are related to the changing landscape of gynaecological surgery itself.

## Competing interests

The authors declare that they have no competing interests.

## Authors' contributions

KKC and DL conceived the idea for the study. EM, FB, KKC, DL and JH designed the questionnaires. EM and PJ analysed the data. The manuscript was written by EM, FB and JH and all the authors approved the final version.

## Pre-publication history

The pre-publication history for this paper can be accessed here:

http://www.biomedcentral.com/1472-6920/11/32/prepub

## Supplementary Material

Additional file 1 **Questionnaire to trainees**. The questionnaire (in a tabulated format) distributed to obstetrics and gynaecology trainees working in the West Midlands region. Responses: # Planning/Started/Completed. † Independently/Primary surgeon/Assistant/Taught. § Now/By ST7/As a consultant/Never. * Yes definitely/Yes/Unsure/No/Definitely not. ^ø ^Very important/important/neither important or unimportant/not very important/not at all important Click here for file

Additional file 2 **Questionnaire to trainers**. The questionnaire (in a tabulated format) distributed to consultants in obstetrics and gynaecology working in the West Midlands region. Responses: # Primary trainer/Secondary trainer/Considering/No. * Yes definitely/Yes/Unsure/No/Definitely not Click here for file
