# Supplementary material for: Is gynaecological surgical training a cause for concern? A questionnaire survey of trainees and trainers
Source: BMC Med Educ. 2011 Jun 13;11:32. doi: 10.1186/1472-6920-11-32 (PMC3146420; doi:10.1186/1472-6920-11-32)
Supplement: Additional file 1 — Questionnaire to trainees. The questionnaire (in a tabulated format) distributed to obstetrics and gynaecology trainees working in the West Midlands region. Responses: # Planning/Started/Completed. † Independently/Primary surgeon/Assistant/Taught. § Now/By ST7/As a consultant/Never. * Yes definitely/Yes/Unsure/No/Definitely not. ø Very important/important/neither important or unimportant/not very important/not at all important [file 1472-6920-11-32-S1.DOC]

**Additional file 1.** Questionnaire to trainees

| What year of training are you in? |  |
| --- | --- |
| How many years have you worked in obstetrics and gynaecology? |  |
| Have you worked in another surgical specialty? For how many years? |  |
| How many deliveries per year does your labour ward currently conduct? | <3000  3000-3999  4000-4999  5000-5999  >6000 |
| How many O&G juniors does your hospital have? | ST1-ST2  ST3-ST5  ST6-ST7+  Staff grades |
| Does the consultant you are currently attached to do: | Obstetrics only  Gynaecology only  Obstetrics and gynaecology (minor surgery only)  Obstetrics and gynaecology (including major surgery) |
| Have you passed: | Part 1 MRCOG  Part 2 MRCOG  MRCS/FRCS |
| Are you planning to undertake a surgical ATSM? | Benign abdominal surgery#  Benign gynaecological surgery: hysteroscopy#  Benign gynaecological surgery: laparoscopy#  Benign vaginal surgery#  Gynaecological oncology#  Urogynaecology# |
| If you have started a surgical ATSM are you on track to complete it? | Yes definitely  Yes  Unsure  No  Definitely not |
| Over the past 8 weeks how many days study/annual/sick leave have you taken? |  |
| Over the past 8 weeks how many gynae theatre sessions have you attended? | Operative list including major cases  Operative list minor cases only  Day case list |
| Over the passed 8 weeks how many cases have you been involved with? | Abdominal hysterectomy†  Vaginal hysterectomy†  Laparoscopic hysterectomy†  Myomectomy†  Diagnostic laparoscopy†  Operative laparoscopy†  Laparoscopic ectopic†  Colposuspension†  Vaginal repair†  Vaginal tape procedure†  Diagnostic hysteroscopy†  Operative hysteroscopy†  Vulval surgery†  Other major procedure†  Other minor procedure† |
| Which of the following procedures do you feel happy to perform independently? | Abdominal hysterectomy§*  Vaginal hysterectomy§*  Laparoscopic hysterectomy§*  Myomectomy§*  Diagnostic laparoscopy§*  Operative laparoscopy§*  Laparoscopic ectopic§*  Colposuspension§*  Vaginal repair§*  Vaginal tape procedure§*  Diagnostic hysteroscopy§*  Operative hysteroscopy§*  Vulval surgery§* |
| Do you think that you are getting enough gynaecological surgical experience? | Yes definitely  Yes  Unsure  No  Definitely not |
| Do you think you are getting enough practical teaching on gynaecological surgery? | Yes definitely  Yes  Unsure  No  Definitely not |
| What do you think are the major barriers to you gaining more gynaecological surgical experience? | Unable to attend theatre sessions due to staff shortages^ø^  Unable to attend theatre sessions due to time off following on calls^ø^  Lack of theatre time to allow training^ø^  Consultant not surgically confident to teach^ø^  Consultant not inclined to teach^ø^  Lack of continuity with the same consultant^ø^ |
| Any other barriers to you gaining surgical experience? | *Free text* |
| Do you attend a theatre session in your own time (days off after on calls/annual leave)? | I attend … sessions  I am prepared to attend … sessions |
| What do you think would improve your surgical skills? | More theatre sessions*  Attachment with a surgical trainer*  Surgical lists specifically designated as training lists*  Attachment to a gynae oncology team*  DVDs demonstrating surgical procedures*  Access to models/laparoscopic trainers*  Access to surgical text books* |
| Any other ways that your surgical skills could be improved? | *Free text* |
| Which procedures do you think step-by-step instructional videos would be helpful for? | Abdominal hysterectomy*  Vaginal hysterectomy*  Laparoscopic hysterectomy*  Myomectomy*  Diagnostic laparoscopy*  Operative laparoscopy*  Laparoscopic ectopic*  Colposuspension*  Vaginal repair*  Vaginal tape procedure*  Diagnostic hysteroscopy*  Operative hysteroscopy*  Vulval surgery* |
| Would instructional videos be helpful for any other procedures? | *Free text* |
| In the future do you see your consultant post containing: | Only obstetrics  Only gynaecology  Obstetrics and gynaecology (minor surgery)  Obstetrics and gynaecology (major surgery)  Undecided |
