# Supplementary material for: Is gynaecological surgical training a cause for concern? A questionnaire survey of trainees and trainers
Source: BMC Med Educ. 2011 Jun 13;11:32. doi: 10.1186/1472-6920-11-32 (PMC3146420; doi:10.1186/1472-6920-11-32)
Supplement: Additional file 2 — Questionnaire to trainers. The questionnaire (in a tabulated format) distributed to consultants in obstetrics and gynaecology working in the West Midlands region. Responses: # Primary trainer/Secondary trainer/Considering/No. * Yes definitely/Yes/Unsure/No/Definitely not [file 1472-6920-11-32-S2.DOC]

**Additional file 2.** Questionnaire to surgical trainers.

| In your consultant practice do you specialise in | Obstetrics only  Obstetrics with minor gynaecological surgery  Obstetrics with major gynaecological surgery  Gynaecology only |
| --- | --- |
| How would you best describe your area of expertise? | Urogynaecology  Infertility  Benign gynaecology  Gynaecological oncology - unit  Gynaecological oncology - centre |
| How many years have you been working at consultant level? | <5 years  5-9 years  10-14 years  15-19 years  >20 years |
| How many deliveries does your unit conduct per year? | <2999  3000-3999  4000-4999  5000-5999  >6000 |
| Which best describes your department’s working pattern? | I have my own team of junior(s)  I share junior(s) with another consultant  I share junior(s) with more than one consultant  There is no team structure |
| Do you have a subspecialty trainee, lecturer or research fellow working with you? | Number of staff |
| Are you a trainer of a surgical ATSM? | Benign abdominal surgery#  Benign gynaecological surgery: hysteroscopy#  Benign gynaecological surgery: laparoscopy#  Benign vaginal surgery#  Gynaecological oncology#  Urogynaecology# |
| How many operating lists do you have per month? (one session = half day) | Lists including major procedures  Lists including minor procedures only |
| Over the past 4 weeks:  Lists including majors  Minors only lists | How many operating sessions have you had?  How many of these lists were attended by trainees?  In total how many different trainees attended these lists? |
| Do you have more than one trainee attending your lists? | Always  Regularly  Rarely  Never |
| What do you think are the major barriers to teaching trainees gynaecological surgery? | Lack of continuity with the same trainee*  Lack of theatre time to allow training*  Cases too complex to allow training*  Operating with a consultant colleague*  Lack of enthusiasm by the trainee to learn*  Trainees lacking basic surgical skills*  Lack of interest by consultants to teach*  Lack of confidence by consultants to teach* |
| Are there any other barriers that prevent trainees gaining gynaecological surgical training? | *Free text* |
| What do you think is the solution to improve gynaecological surgical training? | Trainees to attend lists in their own time*  Increased training on models/laparoscopic trainers*  Attachment to a surgical trainer*  Attachment to a gynae oncology*  Increased team working*  Designated training lists* |
| Are there any other solutions to improving gynaecological surgical training? | *Free text* |
| Do you think OSATS are a genuine measure of competence? | Yes definitely  Yes  Unsure  No  Definitely not |
| Do you think the current programme produces doctors competent in general gynaecological surgery by the end of training? | Yes definitely  Yes  Unsure  No  Definitely not |
| Do you think surgical aptitude testing should be introduced to select trainees for surgical training? | Yes definitely  Yes  Unsure  No  Definitely not |
| Are there any other comments you would like to make about gynaecological surgical training? | *Free text* |
